# Biodegradable double-network GelMA-ACNM hydrogel microneedles for transdermal drug delivery

**DOI:** 10.3389/fbioe.2023.1110604

**Published:** 2023-01-25

**Authors:** Wensheng Lin, Shixian Lin, Xingwu Zhou, Fanwen Yang, Zishan Lin, Shiqing Li, Haoyuan Zhang, Yuehan Ouyang, Jieying Zhu, Wei Sun, Dequn Huang, Baojian Liao, Jixiang Zhu

**Affiliations:** ^1^ The Sixth Affiliated Hospital of Guangzhou Medical University, Qingyuan People’s Hospital, School of Biomedical Engineering, Guangzhou Medical University, Guangzhou, China; ^2^ Department of Pharmaceutical Sciences, University of Michigan, Ann Arbor, MI, United States; ^3^ Key Laboratory of Regenerative Biology, South China Institute for Stem Cell Biology and Regenerative Medicine, Guangzhou Institutes of Biomedicine and Health, Chinese Academy of Sciences, Guangzhou, China; ^4^ Institute of Biological and Medical Engineering, Guangdong Academy of Sciences, Guangzhou, China

**Keywords:** gelatin methacrylate, acellular neural matrix, microneedles, physicochemical cocrosslinking, transdermal drug delivery

## Abstract

As a minimally invasive drug delivery platform, microneedles (MNs) overcome many drawbacks of the conventional transdermal drug delivery systems, therefore are favorable in biomedical applications. Microneedles with a combined burst and sustained release profile and maintained therapeutic molecular bioactivity could further broaden its applications as therapeutics. Here, we developed a double-network microneedles (DN MNs) based on gelatin methacrylate and acellular neural matrix (GelMA-ACNM). ACNM could function as an early drug release matrix, whereas the addition of GelMA facilitates sustained drug release. In particular, the double-network microneedles comprising GelMA-ACNM hydrogel has distinctive biological features in maintaining drug activity to meet the needs of application in treating different diseases. In this study, we prepared the double-network microneedles and evaluated its morphology, mechanical properties, drug release properties and biocompatibility, which shows great potential for delivery of therapeutic molecules that needs different release profiles in transdermal treatment.

## 1 Introduction

Due to a variety of challenges faced by oral drug delivery, such as hepatic first pass effect, the gastric irritation, and poor patient compliance, transdermal drug delivery (TDD) has been used as an alternative strategy for delivering therapeutics (Lee C et al., 2018; Lee et al., 2021). One of the greatest challenges for TDD is overcoming the stratum corneum (SC) that acts as the first protective layer of the skin, which significantly reduce the effectiveness of delivering active ingredients and limit the types of drugs that can be delivered by this route (Lee H et al., 2018). Several enhancement approaches have been developed to increase the permeability of the SC to improve the efficacy of transdermal drug delivery, including using chemical enhancers, iontophoresis, thermal ablation, microdermabrasion, laser and electroporation, but most of these techniques have the risk of damaging skin and require complex devices (Zhang et al., 2019; Kovacik et al., 2020; Ahad et al., 2021; Joshi et al., 2021; Li et al., 2021). These limitations can be overcome by using microneedles (MNs). MNs can deliver a wide range of therapeutic molecules in a minimally invasive manner without damaging neurons in the dermis (Zhu et al., 2020). Compared with the existing percutaneous drug delivery strategies, MNs-mediated transdermal drug delivery facilitates convenient and painless local drug delivery and even achieve self-administration (Chen et al., 2022).

Selection of MNs matrix material could significantly affect the drug loading and release profiles of the encapsulated therapeutics. From a drug release perspective, MNs could be largely classified as burst release and sustained release ones, which mainly depend on the dissolution and degradation properties of the MNs materials within the skin. Natural hydrogels, such as gelatin, cellulose, alginate, chitosan and hyaluronic acid, have been utilized to fabricate MNs for rapid drug release due to their innate water solubility (Xie et al., 2017; Yu et al., 2017; Du et al., 2019; Castilla-Casadiego et al., 2021). In contrast, MNs fabricated by synthetic hydrogel, such as polylactide, polyvinyl alcohol, acrylate polymers, poly (β-ester), and polyvinylpyrrolidone-based MNs, can be further modified by chemical crosslinking for desired drug sustained release profiles (AL-Japairai et al., 2020; Guillot et al., 2020; Lee et al., 2021; Zare et al., 2021; Zhang et al., 2021). However, neither MNs based on synthetic hydrogels nor natural hydrogels allow for a tunable drug release over time to meet the needs of application in different periods and to effectively maintain the activity of the drug during the phased release. For example, in the field of vaccine delivery, the early rapid release of antigen can activate the immune response, and the antibody level can maintain by the subsequent sustained release (Qu et al., 2020; Russell et al., 2022). It is highly desirable to combine the merits of both natural and synthetic hydrogels and to investigate novel materials with innate biocompatibility and biodegradability for versatile drug release control.

Compared to the above materials, acellular matrix (ACM) is distinguished by its ability to store, protect and release therapeutic molecules (Mohiuddin et al., 2020). The hybrid gel network by combining ACM with gelatin methacryloyl (GelMA) to fabricate MNs would be a promising strategy. Specifically, GelMA is a derivative of gelatin and could be crosslinked by ultraviolet (UV) in the presence of photoinitiators to generate chemical-crosslinking network (Liu et al., 2018; Zhou et al., 2020). ACM, as an excellent natural material with high biocompatibility and biodegradability derived from biological tissue, is rich in natural biopolymers including collagens, glycosaminoglycans, proteoglycans, and glycoproteins, which can induce sol-gel transition by a process of physical self-assembly (Saldin et al., 2017). There are reports showing that tissue-specific biologic materials have properties that enhance better site-appropriate cellular adhesion, proliferation, and differentiation compared to ACM derived from non-homologous tissue sources (Medberry et al., 2013; Pattar et al., 2019). Therefore, the choice of ACM could be determined by the type of drug loaded and corresponding applications. We aim to develop a fabrication strategy with modularity in selecting different ACM and the versatility in loading various biomolecules to meet the needs of application in different diseases. Here, we chose acellular neural matrix (ACNM) as the carrier of calcitonin gene-related peptide II (CGRP II), which play an important role in chronic neuropathic pain, verifying the feasibility of the proposed method conceptually (Xie et al., 2017; Khan et al., 2019; de Vries et al., 2020).

**FIGURE 1:**
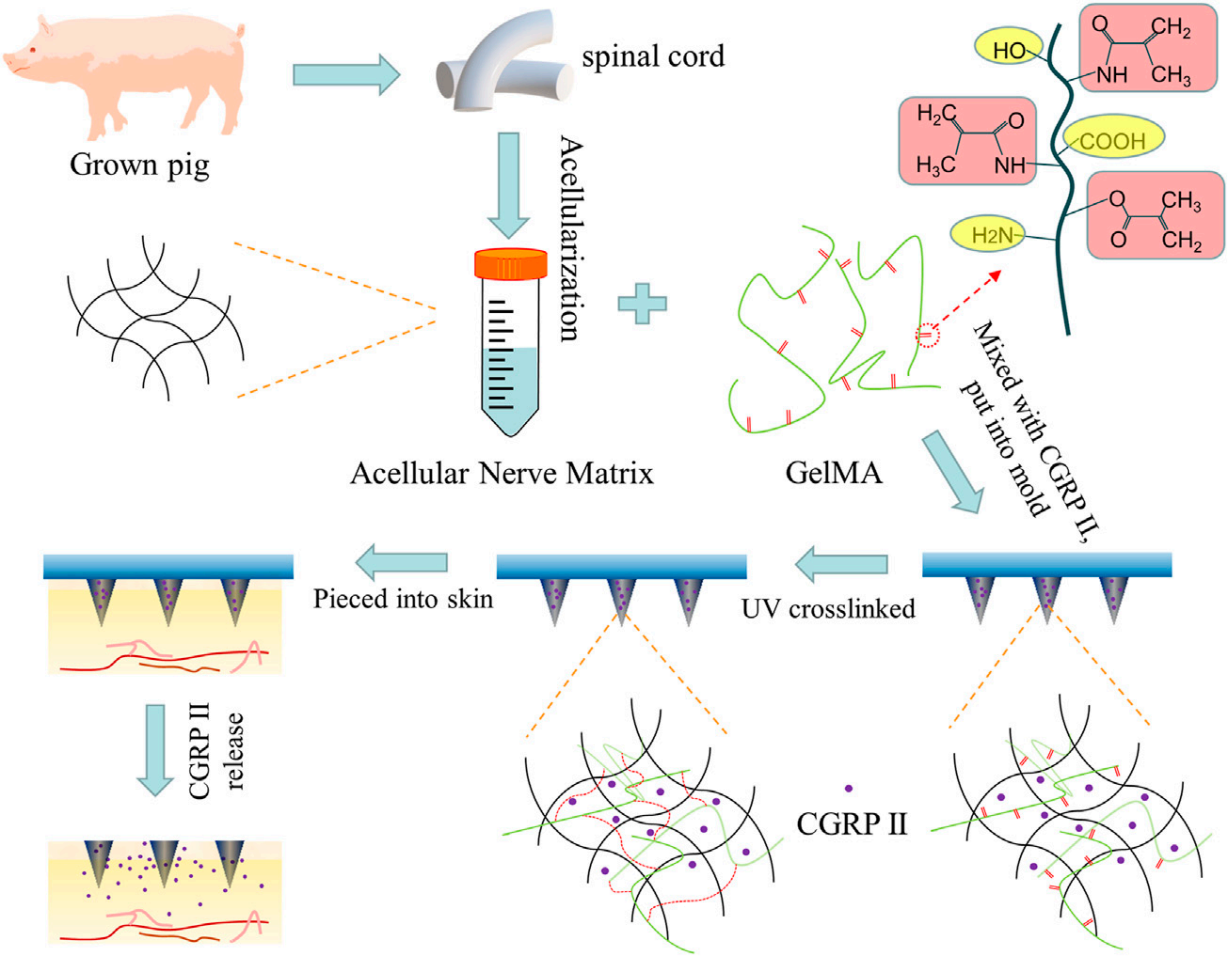
Scheme of the design of biodegradable microneedle patch fabricated by double-network GelMA-ACNM hydrogel for transdermal drug delivery.

Specifically, we designed a double-network MNs (DN MNs) that combined ACNM, as an early drug release matrix, and GelMA for sustained release (Figure 1). After the DN MNs application, the tips can be left inside the skin, along with a rapid and prolonged release of the therapeutic molecules. This is a novel strategy to meet the needs of application in different diseases without complex devices required and maintains the activity of the drug to meet therapeutic requirements. In this study, the release behaviors of the DN MNs were observed in the agarose hydrogel that acted as an artificial skin model and further determined with Enzyme Immunoassay (EIA). The DN MNs exhibits a two phases release: rapid release phase and sustained release phase. We also verify the biocompatibility of the DN MNs *in vivo* studies. As a result, our DN MNs presents a potentially valuable method for delivery of therapeutic molecules that needs different release profiles in transdermal treatment.

## 2 Materials and methods

### 2.1 Materials

Gelatin (porcine skin) and Rhodamine B was purchased from Aladdin (Shanghai, China). Photoinitiator (2-hydroxy-4′-(2-hydroxyethoxy)-2-methylpropiophenone), Agarose (≥1,200 g/cm^2^) and Pepsin (1:3000) was purchased from Acmec Biochemical Co., Ltd. (Shanghai, China). Methacrylic anhydride (94%) were purchased from Sigma-Aldrich (Shanghai, China). Dialysis bag (12–14 kDa) was purchased from Yuanye Bio-Technology Co., Ltd. (Shanghai, China). Phosphate buffer (PBS) solution was self-prepared, and the other materials were purchased from Wuhan Servicebio Technology Co., Ltd. (Wuhan, China).

### 2.2 Preparation of the ACNM

The decellularization process of spinal cord tissues (derived from adult pig) were first agitated in the following decellularization baths sequentially: deionized water (6 h at 4°C, 60 rpm), 0.02% trypsin/0.05% EDTA (1 h), 3.0% Triton X100 (12 h), deionized water (rinse 4 times), 4% SDS solution (24 h, 60 rpm), deionized water (rinse for 1 h), 4% SDS solution (24 h, 60 rpm), deionized water (rinse for 1 h). Subsequently, the decellularized ACNM were dissolved with pepsin (1.0 mg/ml) in HCl (0.01 M) at a concentration of 10 mg/ml after lyophilized for 48 h. Finally, the solution was stirred at room temperature for 48 h and neutralized to pH 7.4 with 0.1 M NaOH to form a ACNM hydrogel.

### 2.3 Synthesis of GelMA

10% (w/v) gelatin solution was made by dissolving 20 g gelatin in 200 ml preheated PBS at 50°C for 1 h. After gelatin fully dissolved, 16 ml of methacrylic anhydride was slowly added to the gelatin solution and stirred at 50°C for 2 h. Subsequently, the reaction was stopped by an additional 200 ml of PBS was added and the solution was dialyzed for 7 days at 40°C to remove impurities by using dialysis bag with a molecular weight cut-off of 12–14 kDa. The dialyzed product was filtered and frozen to −20°C in 50 ml conical tubes for lyophilization. After 3 days of lyophilization, GelMA was obtained and stored with airtight and light-free until further use.

### 2.4 Preparation and characterization of ACNM-GelMA hydrogel

2.5 g of GelMA was dissolved in 10 ml of ACNM (1%, w/v) to prepare the GelMA-ACNM hydrogel. The morphology of the GelMA-ACNM hydrogel was investigated through a scanning electron microscope (ProX, Phenom, Netherlands). The GelMA-ACNM hydrogel was freeze-dried and sputtered with gold before observing by SEM. The effective amounts of intact CGRP II before and after UV irradiation by using EIA. “Before UV irradiation”: 40.0 μg CGRP II was dissolved in 200 μl GelMA-ACNM hydrogel. “After UV irradiation”: above hydrogels were irradiated by UV (270 mW/cm^2^) for 30 s. The stability of CGRP II in GelMA-ACNM hydrogel were further evaluated. “Original”: 40 μg CGRP II was dissolved in 200 μl GelMA-ACNM hydrogel. “1, 4, 7 day”: above hydrogels were stored at 26°C for 1, 4, 7 day. As a control, 40 μg CGRP II was dissolved in 200 μl PBS and stored at 26°C for 1, 4, 7 day. Each sample consisted of three replicate measurements and the results was expressed as an average value.

### 2.5 Fabrication of the MNs

The MNs were fabricated according to a two-step template replication method. Specifically, 20% (w/v) gelatin was dissolved in deionized water as the backing layer solution; 2.5 g of GelMA, 50 mg of ultraviolet (UV) photoinitiator were dissolved in 10 ml of PBS solution to prepare the GelMA hydrogel; 2.5 g of GelMA, 50 mg of UV photoinitiator were dissolved in 10 ml of ACNM (1%, w/v) to prepare the GelMA-ACNM hydrogel. To load Rhodamine B or CGRP II, 5 mg Rhodamine B or 2 mg CGRP II was dissolved in above hydrogel solution as the tip material. Then, 200 μl of the tip solution was poured over a female polydimethylsiloxane (PDMS, Henan Micro-Nano BenTeng Biotechnology Co., Ltd., China) mold and remove the bubbles up the MN tips by vacuumize, and then, centrifugation at 3,500 rpm for 10 min to draw the tip solutions filled the cavities of the template. After removing the redundant solution and solidifying the tips by UV irradiation (270 mW/cm^2^) for 30 s, the solution of backing layer material was added to cover the tips and dried at room temperature overnight. Finally, the patch named as CN MNs (chemical network) and DN MNs (double-network) was peeled off from the mold. For PN MNs (physical network), 2.5 g of GelMA was dissolved in 10 ml of ACNM (1%, w/v), the rest of procedures were similar to the preparation of the DN MNs but without UV irradiation.

### 2.6 SEM characterization

The morphology of the DN MNs was investigated through a scanning electron microscope (ProX, Phenom, Netherlands). The MNs were sputtered with gold before observing by SEM.

### 2.7 Mechanical property tests

The mechanical strength of the dry MNs was tested by a universal tensile compression tester (UTM4304X, Sansi Test Equipment Co., Ltd., China). MNs were placed on a fixed station with their tips facing upward. Under a constant 2 mm/min speed of force sensor, and the maximum loading force was set to 5 N, the mechanical properties of MNs were profiled. For wet MNs mechanical strength test, the MN tips were first immersed in PBS solution for 10 s. The rest of the procedure is the same as the dry MNs procedure.

### 2.8 Release properties of the MNs *in vitro*

Agarose hydrogel was used to simulate the skin to investigate the Rhodamine B release properties of the CN MNs, PN MNs and DN MNs. The agarose hydrogel (1%, w/v) was first prepared by dissolving agarose powder in boiled deionized water and cooling. The MNs were then pressed to the surface of the agarose hydrogel for 7 days. Finally, cut the agarose hydrogel to capture the depth wise color change images by cameras every day and analyzed with Photoshop software. Furthermore, The CGRP II release test was determined with CGRP II - EIA Kit (MeiMian Industrial Co., Ltd., Jiangsu, China). The MN patches was first immersed in 2 ml PBS at 37°C, and then, all of release medium was collected at pre-set intervals and replaced with the same amount of fresh PBS. The CGRP II concentration of each release medium was measured with the EIA according to the standard protocol of the product. In addition, the morphology of the MNs with different time points (0, 2 and 7 day) was observed with an optical microscope by incubating the MNs in PBS at 37°C.

### 2.9 *In vivo* biocompatibility of the MNs

All the animal work was approved by the Animal Care Committee of Guangzhou Medical University and conducted in alignment with relevant guidelines. Female Sprague-Dawley rats (150–200 g body weight) were purchased from the Guangdong Medical Laboratory Animal Center and acclimatized for 1 week in an approved animal facility.

DN MNs were inserted into the dorsal skin of the rat for 5 min and 24 h and the animals were euthanized by cervical vertebra dislocation. Skin tissue subject to MNs application was removed and immersed in 4% paraformaldehyde. The fixed tissues were then dehydrated, embedded in paraffin, and made into serially sections for further histology and immunofluorescent staining. Briefly, the fluorescent images were imaged using a Zeiss inverted microscope after the paraffin-embedded skin tissues were cut into 8-μm-thick sections and stained with H&E staining. For fluorescence images, a series of skin tissues sections were deparaffinized and underwent heatinduced antigen retrieval using citrated buffer according to the standard protocol. After immersed in antigen retrieval buffer, permeabilized in 0.3% Triton PBST, and blocked with goat serum for 30 min, sections were incubated with primary antibody against CD68 (rat, 1:200; Abcam) overnight at 4°C. Then, the sections rinsed with PBST and incubated with Alexa 555-conjugated secondary antibody at a dilution of 1:1000 for 60 min at room temperature. After incubation, the sections were counterstained with DAPI for 5 min and the fluorescent images were captured under a Zeiss inverted microscope.

### 2.10 Statistical analysis

The statistical analysis was performed by SPSS software version 25.0. Experiments were run in triplicate unless stated otherwise. All the data presented in this study were expressed as mean ± standard deviation (SD). Statistical analysis was performed using Student’s *t*-test. *p* < 0.05 were considered statistically significant (*p < 0.05, **p < 0.01).

## 3 Results and discussion

### 3.1 Preparation and characterization of ACNM-GelMA hydrogel

**FIGURE 2:**
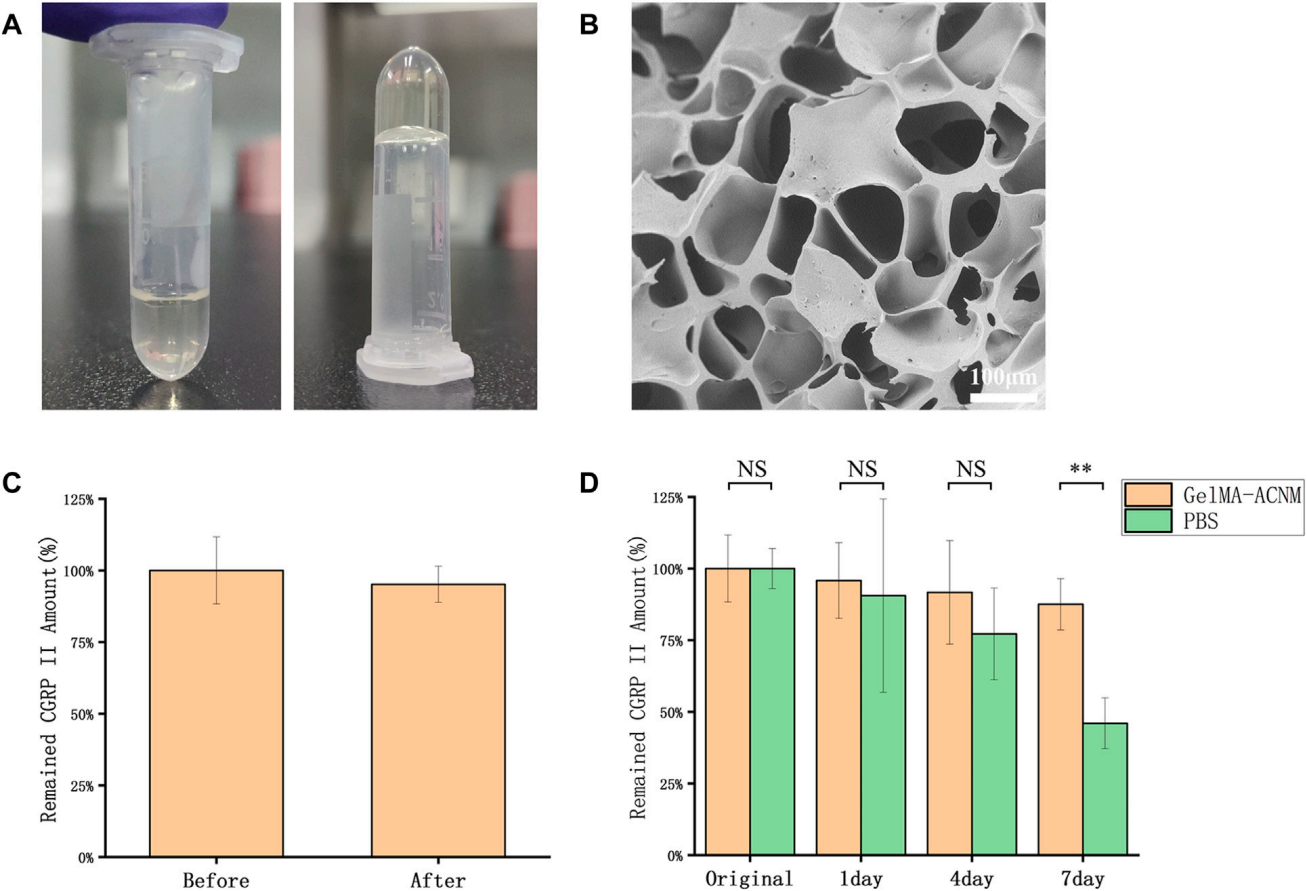
ACNM-GelMA characterization. **(A)** Optical images of the ACNM-GelMA. **(B)** SEM image of ACNM-GelMA. **(C)** The effective amounts of intact CGRP II before and after UV irradiation, determined with EIA. **(D)** The stability of CGRP II peptides retained in the GelMA-ACNM hydrogel, determined with EIA. The data are expressed as the means ± SD (*n* = 3). *p < 0.05, **p < 0.01.

The microstructure and biological functions of ACNM-GelMA hydrogel were analyzed. After UV irradiation, the gel can be rapidly formed by cross-linking (Figure 2A). The SEM showed a porous structures of ACNM-GelMA (Figure 2B). Consistent with other reports, our study found that the Young’s modulus of the hydrogel is higher at higher concentrations (Supplementary Figure S1) (Zhang et al., 2020; Zhu et al., 2020). Although the increase in concentration contributes to the improved mechanical strength, it is difficult to remove air bubbles during the preparation of microneedles if the concentration of GelMA-ACNM was over 25%. Thus, the ACNM-GelMA concentration was selected as 25% in the following experiment. In addition, we investigated the effect of UV irradiation on drug activity by loading microneedles with CGRP II, a neuro-peptide synthesized and released by neurons, which acts as a potent vasodilator as well as a neurotransmitter for the development and maintenance of neuropathic pain states (Edvinsson, 2017; Xie et al., 2017; Wattiez et al., 2020; Edvinsson, 2021). As shown in Figure 2C, 95.2 ± 6.3% of the CGRP II peptide remained stable after UV irradiation. This result indicates that GelMA-ACNM hydrogel successfully carries and protects the CGRP II peptide. The stability of CGRP II in GelMA-ACNM hydrogel were further evaluated *via* EIA. As shown in Figure 2D, the remaining CGRP II amount of GelMA-ACNM hydrogel stored at 26°C for 1, 4 and 7 days are 95.8% ± 13.2%, 91.7% ± 18.1%, and 87.5% ± 9.0%, respectively. In contrast, the remained CGRP II amount of PBS are 90.5% ± 33.7%, 77.2% ± 17.0%, and 46.0% ± 8.9%, respectively. Major activity of CGRP II was effectively maintained within GelMA-ACNM hydrogel under general storage conditions, which indicates the advantages of GelMA-ACNM in maintaining drug activity to meet the needs of application in different periods.

### 3.2 MNs fabrication and characterization

**FIGURE 3:**
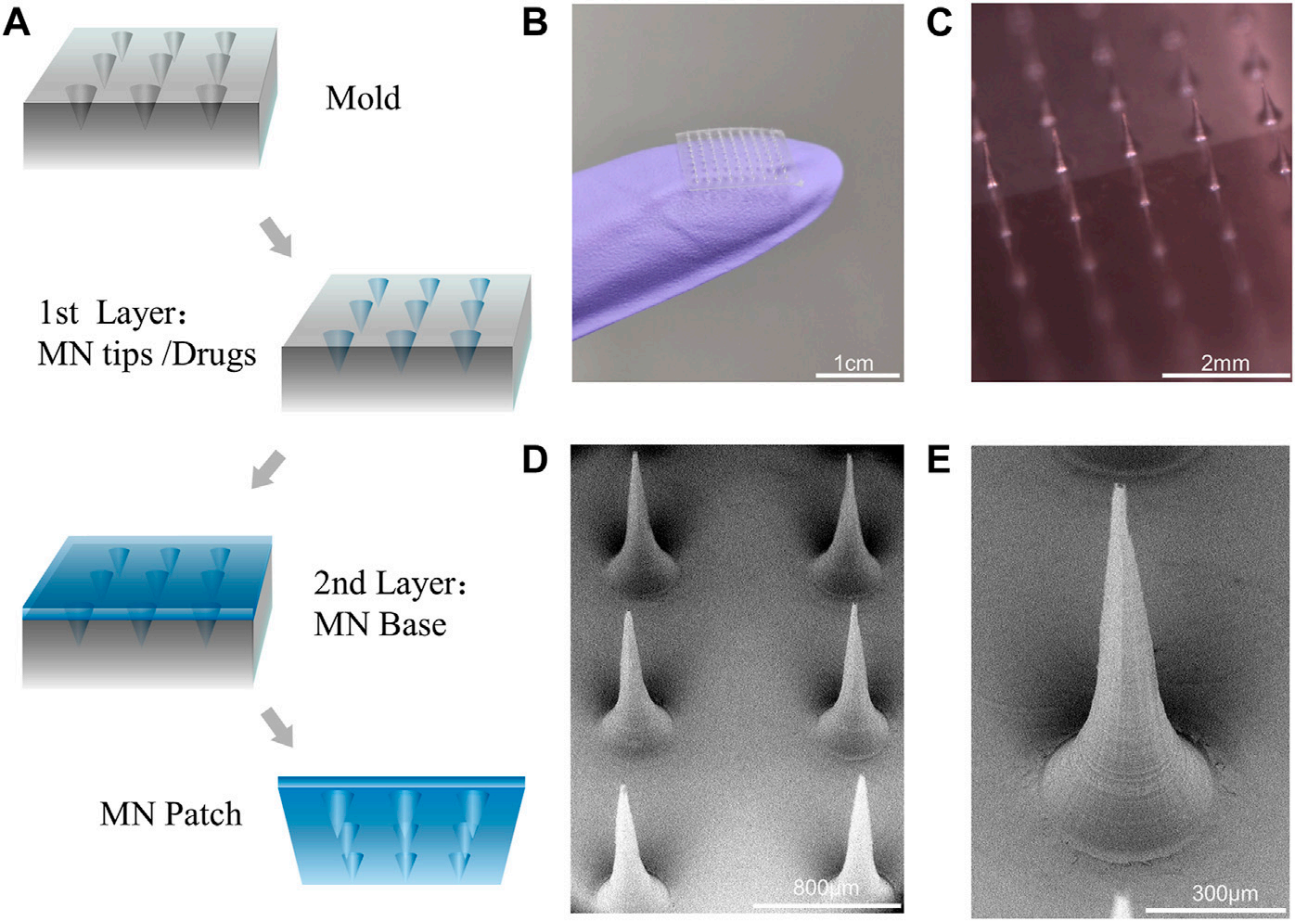
MNs fabrication and characterization. **(A)** Scheme of the design of MNs by a two-step template replication method. **(B,C)** Optical images of the MNs. **(D,E)** SEM image of MNs and magnified MN tips.

In a typical experiment, the DN MNs were fabricated *via* a two-step template replication method (Figure 3A). During this process, the DN MNs template was first loaded with a predetermined amount of GelMA-ACNM solution, and the bubbles were removed by vacuum. Subsequently, tip material was filled into MN mold cavities by centrifugation to form MN tips. After removing the redundant solution and solidifying the tips after UV irradiation, the solution of backing layer material, gelatin, was added to cover the tips and dried at room temperature overnight. Finally, the MNs could be obtained by demolding. As shown in Figures 3B–E, arrays of 9 × 9 needles were obtained over a square array of 13.5 mm patch, with a height of 720 microns and a bottom diameter of 370 microns of each MNs appearing tapered in shape. A two-step template replication method under mild fabrication conditions for MNs preparation is critical for its ability to deliver temperature sensitive drugs and prevent agglomerates formation along the structure. As shown in Supplementary Figures S2, S3, the MNs contains about 5.5 ± 0.9 μg CGRP II and the drug was concentrated on the microneedle tips with uniform distribution. Drug loading of only the tip of the MNs is an advantage, which can improve the utilization rate of the drug, facilitate long-term treatment, and reduce the risk of medical waste misuse.

### 3.3 Mechanical property of the MNs

**FIGURE 4:**
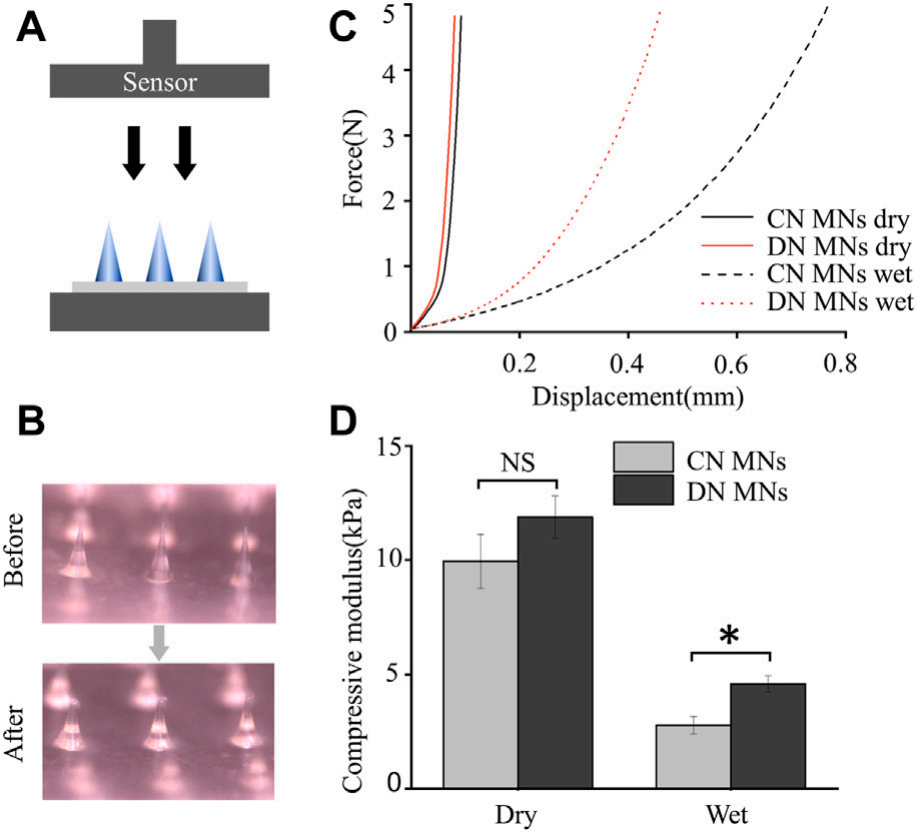
Mechanical property of the chemically crosslinked network MNs (CN MNs) and double-network MNs (DN MNs). **(A)** Schematics of the setups for testing mechanical strength. **(B)** Optical images of the dry MNs before and after the mechanical strength test. **(C)** The mechanical strength properties of the MNs. For wet test, the MN tips were first immersed in PBS solution for 10 s. **(D)** Quantification of the mechanical strength. The data are expressed as the means ± SD. (*n* = 3). *p < 0.05.

To evaluate mechanical property of the DN MNs, we determined the axial force load versus displacement of the DN MNs using a compression method and compared them with the chemically crosslinked MNs (CN MNs). During the test, the MNs were first placed on a horizontally positioned glass slide with tips facing a force sensor that slowly approached the MNs. Then the force measurements started when the sensor touched the MN tips and lasted until it applied force 5 N (Figure 4A). Mechanical strength results showed that no discontinuity in the applied force along with the displacement, which indicates that these needles did not fracture, but instead bent (Figures 4B, C). The DN MNs and CN MNs showed similar compressive moduli without significant differences in dry conditions. The physical properties of the wet MNs were further observed. The results showed that the DN MNs was stronger than CN MNs, a phenomenon that might be attributed to the mechanical stiffness enhanced by a second physical network (Figure 4D). The data above indicated that the DN MNs was endowed with appropriate mechanical properties for transdermal drug delivery.

### 3.4 Release behaviors of the MNs *in vitro*

**FIGURE 5:**
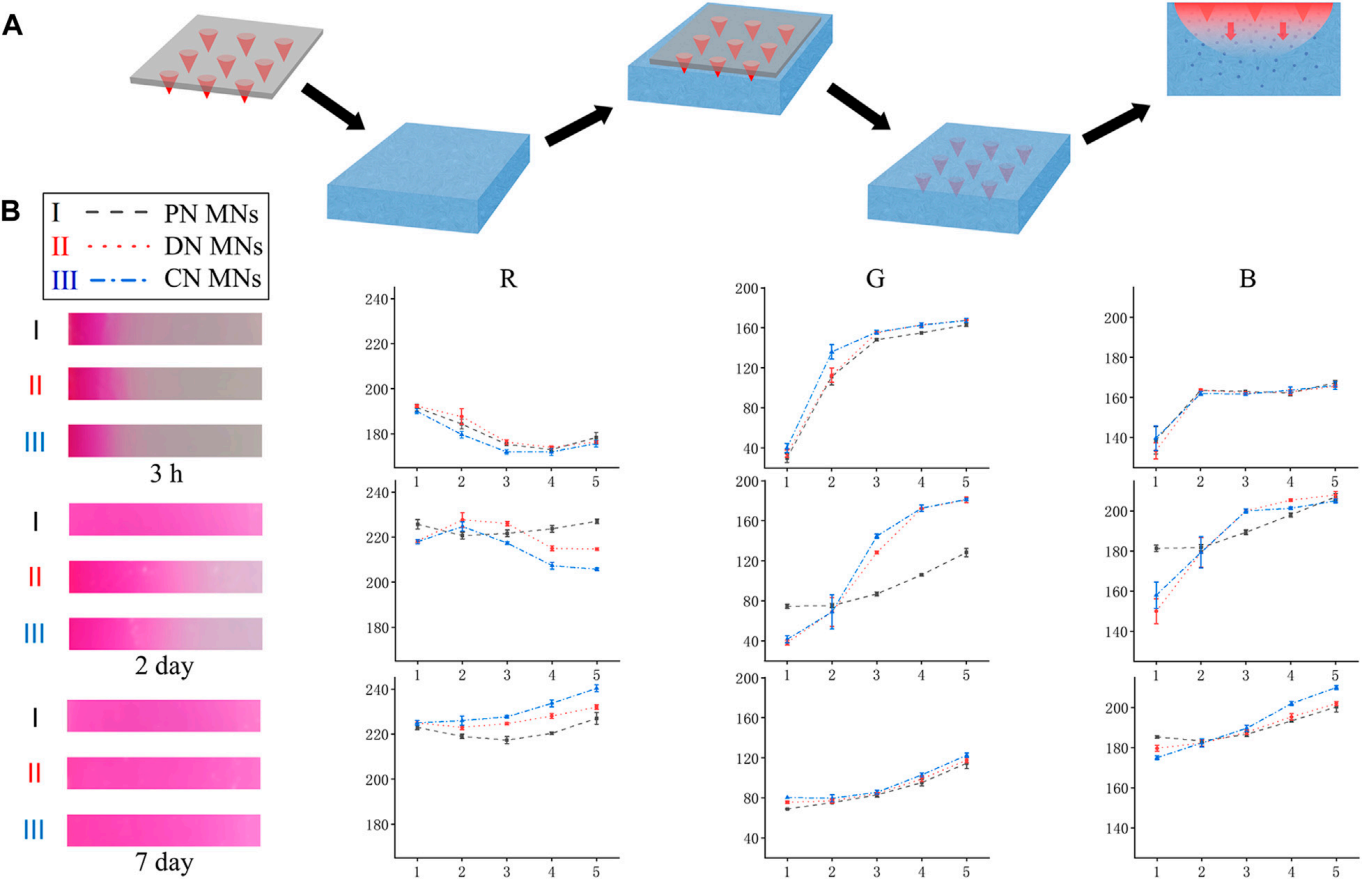
*In vitro* release of Rhodamine B from the MNs into the agarose hydrogel that acted as a skin model. **(A)** Schematic for the vitro release design. **(B)** Left: Snapshot showing the depthwise color change in the cross section of the agarose hydrogel after Rhodamine B (10 mg/ml) release from the microneedles over time. Right: Line graph of RGB color change of scanned image, analyzed with Photoshop software. X-axis represents the grey level at the corresponding position, and Y-axis indicates the grey levels of each color channel (Red, Green, Blue). PN MNs: physically crosslinked network MNs. Points represent means ± SD. (*n* = 3).

To evaluate the drug release profile, a preliminary test was performed on the agarose hydrogel that acted as an artificial skin model (Figure 5A) (Chang et al., 2017). MNs were loaded with a fluorescent dye, Rhodamine B, for better visualization. During the vitro tests, three different MNs, that is, the CN MNs that were chemically crosslinked network, the PN MNs that were physically crosslinked network, and the DN MNs that were double-network, were first prepared and were pressed into the agarose hydrogel. The changes of the depthwise color in the cross section of the agarose hydrogel were measured at specific time points. It was observed that all three groups generated color change visible to the naked eye in the first 3 h without significant differences. However, when the release time increased, the color of PN MNs group diffused at a high speed, and the area was completely covered with agarose within 2 days, which was significantly larger than that of CN MNs and DN MNs groups (Figure 5B). The RGB value, which provide quantification information of the color display were further analyzed. The results showed that the RGB value dropped with a shifting rate of PN MNs > DN MNs > CN MNs, especially in R and G channels.

**FIGURE 6:**
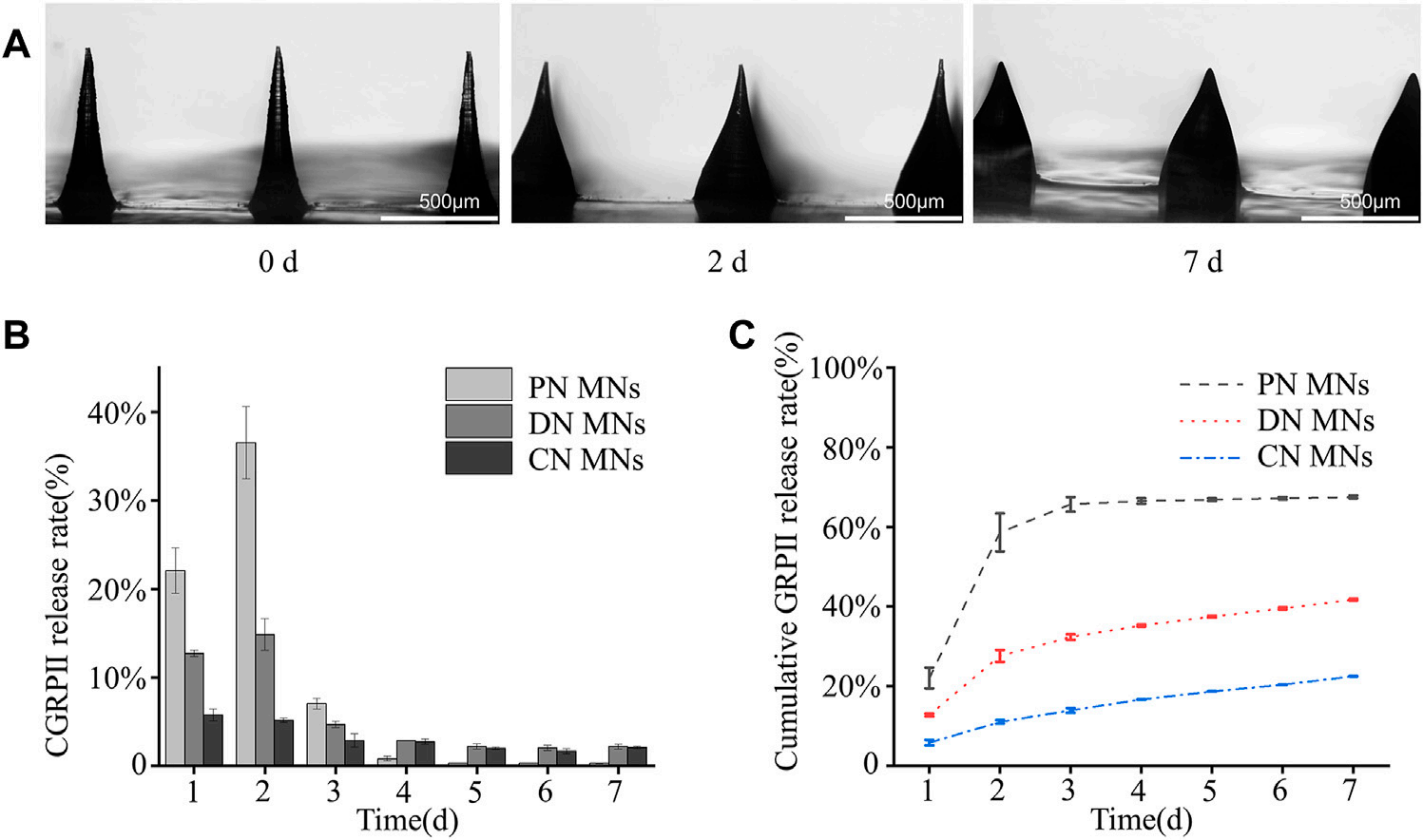
Degradation of the MNs and CGRP II release. **(A)** Optical images of the degradation of the DN MNs in PBS solution for different time points (0, 2 and 7 day). **(B)** The release profiles of CGRP II from different MNs in PBS solution at 37°C. **(C)** Cumulative CGRP II release *in vitro* from different MNs in PBS solution at 37°C, shown as a function of time. Points represent means ± SD. (*n* = 3).

In addition, the degradation of the DN MNs was measured by incubating them in PBS solution. As shown in Figure 6A, it was observed that the swelling of the DN MNs and the needle tip height reduction after the immersion of PBS solution, which might be explained by the fact that the whole system absorbs water and the relatively high solubility of the DN MNs with physical crosslinking led to partial dissolution. Notably, swelling effects of the MNs could also facilitate the release of the drug (Luo et al., 2019; Chew et al., 2020). As shown in Supplementary Figure S4, all three types of MNs exhibited high swelling rates after immersion in PBS (PN MNs > DN MNs > CN MNs), and PN MNs would be completely dissolved within 30 min due to the lack of chemical crosslinking network. Furthermore, we detected the release kinetics of CGRP II using release media of PBS solution. In the first 3 days, a cumulative release of the CGRP II quickly reached round 80% for PN MNs while only 20% of CGRP II for CN MNs was released after a 7 days incubation (Figures 6B, C). Meanwhile, CGRP II from DN MNs released at a high rate in the first 2 days and constantly released for the rest of the week, exhibiting a fast release with a prolonged release combined. As a novel drug delivery approach, the biphasic drug delivery could meet the needs of optimal treatment requirement, thus improving therapeutic efficacy in the treatment (Battisti et al., 2019; Park et al., 2019). In comparison with other drug delivery systems with complexed fabrication process and requirement of dedicated devices (Yeung et al., 2019; Wang et al., 2022), physicochemical co-crosslinked DN MNs is an effective and convenient way to achieve tunable drug release properties.

### 3.5 *In vivo* biocompatibility of DN MNs

**FIGURE 7:**
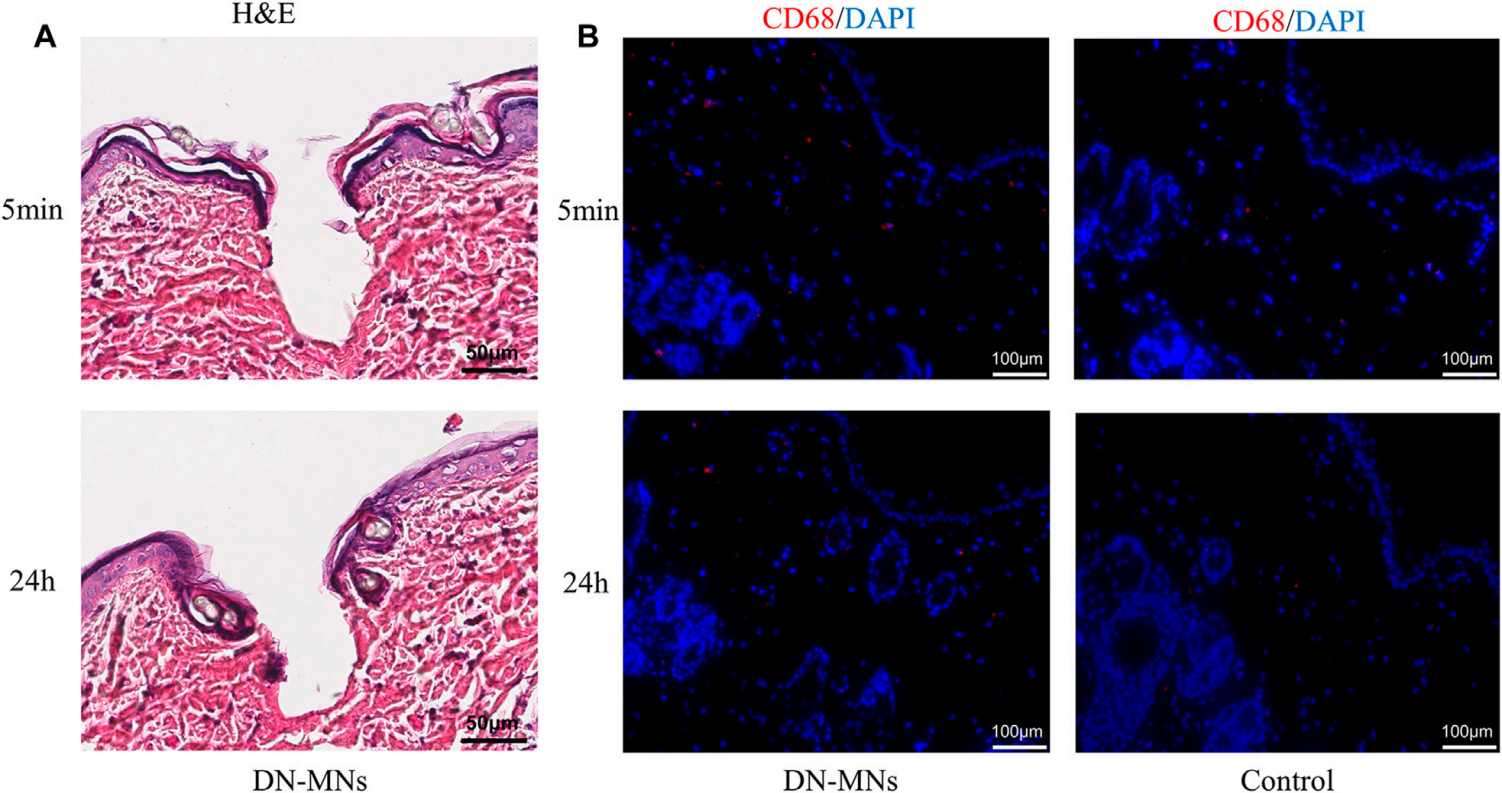
*In vivo* biocompatibility of DN MNs. **(A)** H&E staining of skin tissues after DN MNs application at 5 min and 24 h post-implantation. **(B)** Immuno-histological staining of immune cells in the tissue.

To investigate the biocompatibility of the DN MNs, we assessed the topical application of the DN MNs to the Sprague-Dawley rat skin. The DN MNs were applied to the dorsal skin of the rat and the rat were then sacrificed at 5 min and 24 h for histological and immunofluorescence analysis. As shown in Figure 7A, DN MNs effectively penetrated the epidermal layer of the skin, and no inflammation was observed in the localized area at the site of MNs application. Immunofluorescence staining was further performed to evaluate any signs of inflammation. Compared to the control group, no significant differences of mononuclear cell (CD68+ macrophage) infiltration were observed in DN MNs group, as displayed in Figure 7B. Our results show that DN MNs are capable of penetrating the skin for drug delivery and are biocompatible with the skin.

## 4 Conclusion

In this study, we demonstrated that double-network GelMA-ACNM hydrogels can be used for the fabrication of a DN MNs to achieve a tunable drug release profile over time. We also showed that the DN MNs exhibited sufficient mechanical strength to penetrate the skin, and the DN MNs released their loaded therapeutic agents through both swelling and degradation of the MNs tips. Compared with sustained release for a long period by using some synthetic materials, the DN MNs exhibited a two-phase release: rapid release phase and sustained release phase, which could meet the treatment requirement of certain diseases at the early stages. As a minimally invasive platform, the DN MNs has the potential to be further optimized for different applications. We envisioned that developing a fabrication strategy with modularity in selecting different ACM and the versatility in loading various biomolecules could meet the needs of its application in treating different diseases. In comparison with other drug delivery systems fabricated through complexed processes with dedicated devices, physicochemical co-crosslinked DN MNs is an effective and convenient way to achieve tunable drug release.

## Data Availability

The raw data supporting the conclusion of this article will be made available by the authors, without undue reservation.
