# Supplementary material for: Biodegradable double-network GelMA-ACNM hydrogel microneedles for transdermal drug delivery
Source: Front Bioeng Biotechnol. 2023 Jan 25;11:1110604. doi: 10.3389/fbioe.2023.1110604 (PMC9905680; doi:10.3389/fbioe.2023.1110604)
Supplement: Supplementary file 1 [file DataSheet1.docx]

Supplementary Material

## Supplementary Figures

A

B

**Supplementary Figure 1.** **(A)** The mechanical strength properties of the ACNM-GelMA hydrogel. (D) Quantification of the mechanical strength. The data are expressed as the means ± SD. (n = 3).

**Supplementary Figure 2.** The effective amounts of intact CGRP II before and after MNs fabrication, determined with EIA. The data are expressed as the means ± SD (n = 3).

A

B


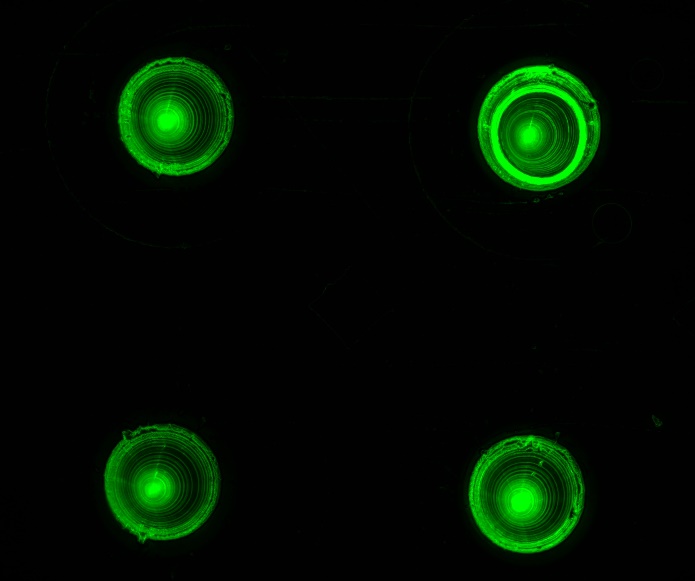

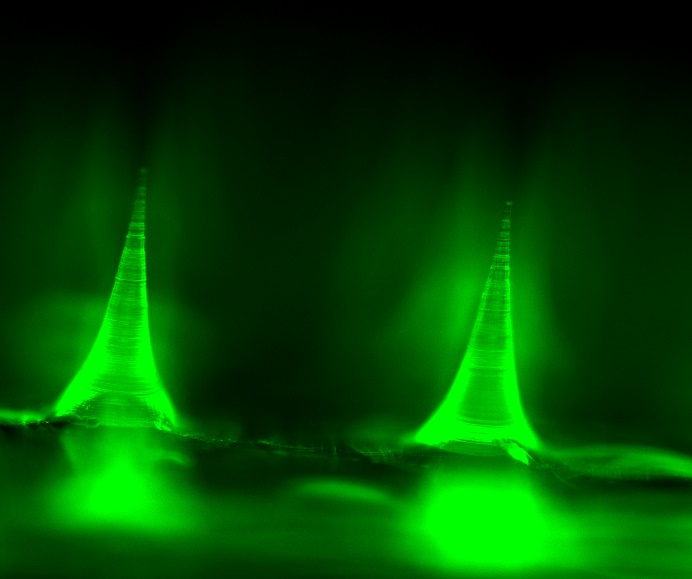


**Supplementary Figure 3.** Fluorescence microscopy image of an MNs loaded with FITC-BSA. **(A)** Images of the backing layer of MNs. **(B)** Images of the MN tips.


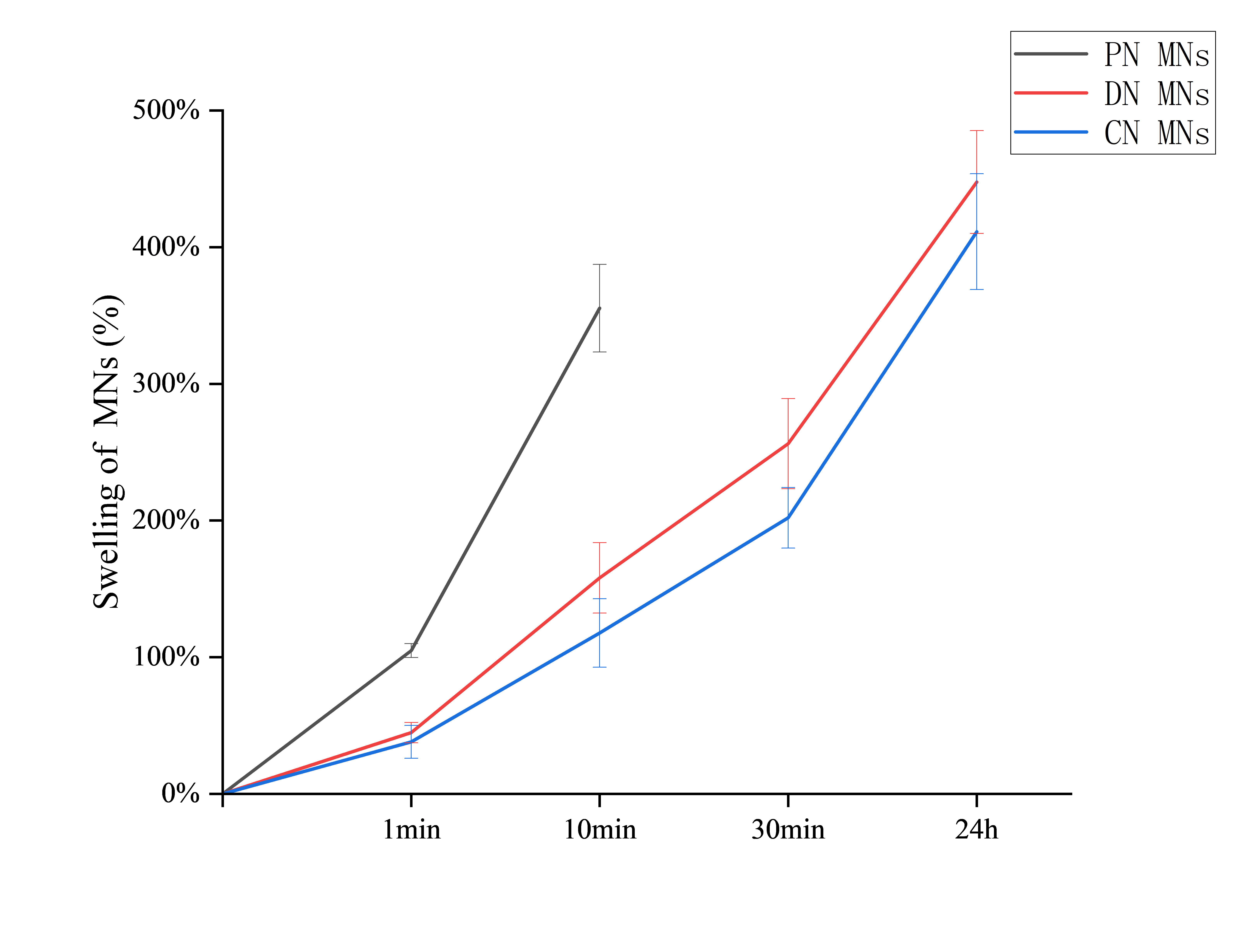

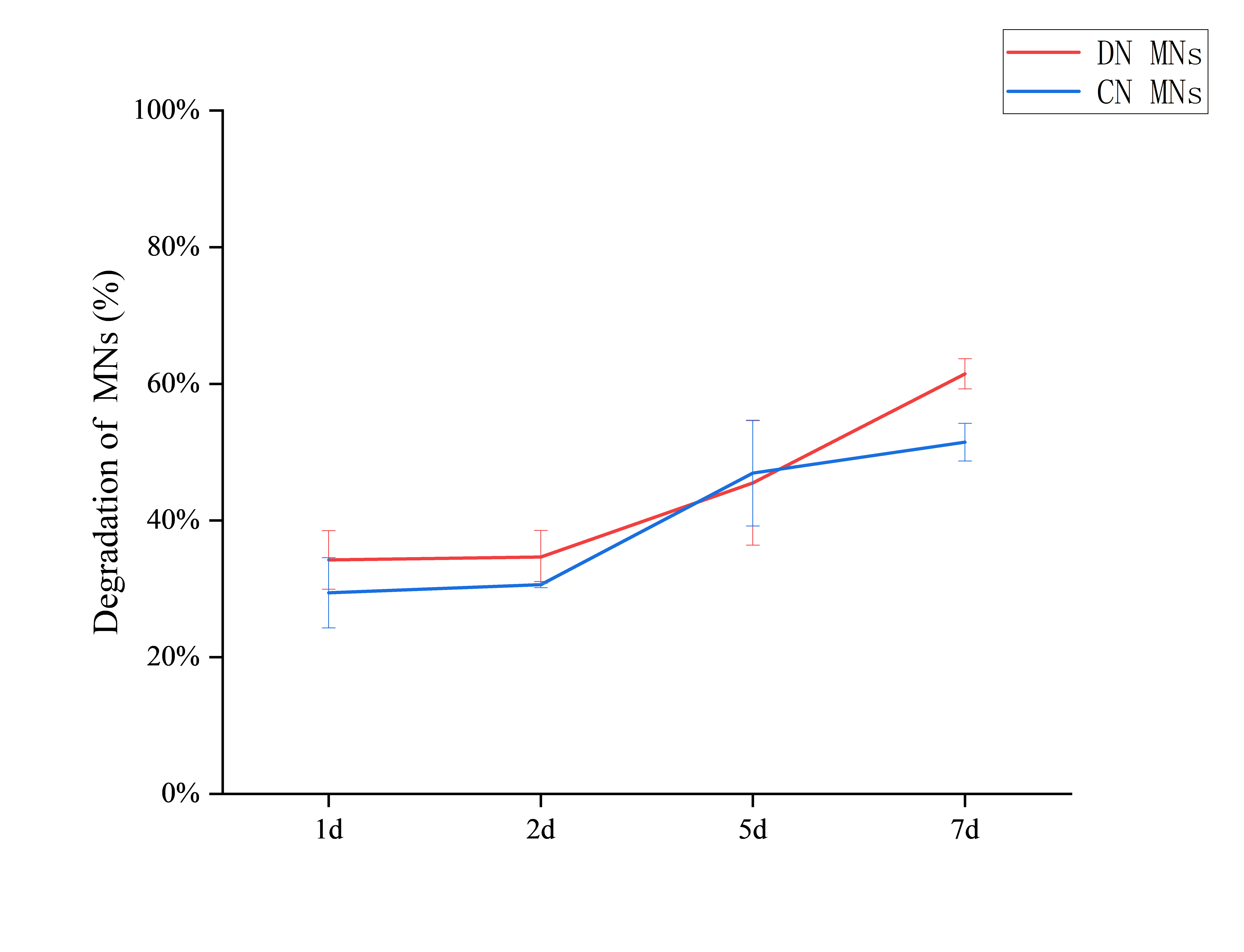


A

B

**Supplementary Figure 4.** Characterization of the swelling and degradation properties of MNs. **(A)** Swelling rate of three types of MNs, PN MNs would be completely dissolved within 30 min. **(B)** Degradation properties of DN MNs and CN MNs. The data are expressed as the means ± SD (n = 3).
